# Biofabrication of a shape-stable auricular structure for the reconstruction of ear deformities

**DOI:** 10.1016/j.mtbio.2021.100094

**Published:** 2021-01-21

**Authors:** I.A. Otto, P.E. Capendale, J.P. Garcia, M. de Ruijter, R.F.M. van Doremalen, M. Castilho, T. Lawson, M.W. Grinstaff, C.C. Breugem, M. Kon, R. Levato, J. Malda

**Affiliations:** a Department of Orthopaedics, University Medical Center Utrecht, Heidelberglaan 100, Utrecht, 3584 CX, the Netherlands; b Department of Plastic, Reconstructive and Hand Surgery, University Medical Center Utrecht, Utrecht, the Netherlands; c Regenerative Medicine Center Utrecht, Utrecht, the Netherlands; d Robotics and Mechatronics, Faculty of Electrical Engineering, Mathematics & Computer Science, University of Twente, Enschede, the Netherlands; e Bureau Science & Innovation, Deventer Hospital, Deventer, the Netherlands; f Departments of Chemistry and Biomedical Engineering, Boston University, Boston, USA; g Department of Plastic, Reconstructive and Hand Surgery, Amsterdam University Medical Center, Emma Children's Hospital, Amsterdam, the Netherlands; h Department of Clinical Sciences, Faculty of Veterinary Science, Utrecht University, the Netherlands

**Keywords:** Bioprinting, Auricular cartilage, Mechanical reinforcement, Shape preservation, Cartilage progenitor cells

## Abstract

Bioengineering of the human auricle remains a significant challenge, where the complex and unique shape, the generation of high-quality neocartilage, and shape preservation are key factors. Future regenerative medicine–based approaches for auricular cartilage reconstruction will benefit from a smart combination of various strategies. Our approach to fabrication of an ear-shaped construct uses hybrid bioprinting techniques, a recently identified progenitor cell population, previously validated biomaterials, and a smart scaffold design. Specifically, we generated a 3D-printed polycaprolactone (PCL) scaffold via fused deposition modeling, photocrosslinked a human auricular cartilage progenitor cell–laden gelatin methacryloyl (gelMA) hydrogel within the scaffold, and cultured the bioengineered structure *in vitro* in chondrogenic media for 30 days. Our results show that the fabrication process maintains the viability and chondrogenic phenotype of the cells, that the compressive properties of the combined PCL and gelMA hybrid auricular constructs are similar to native auricular cartilage, and that biofabricated hybrid auricular structures exhibit excellent shape fidelity compared with the 3D digital model along with deposition of cartilage-like matrix in both peripheral and central areas of the auricular structure. Our strategy affords an anatomically enhanced auricular structure with appropriate mechanical properties, ensures adequate preservation of the auricular shape during a dynamic *in vitro* culture period, and enables chondrogenically potent progenitor cells to produce abundant cartilage-like matrix throughout the auricular construct. The combination of smart scaffold design with 3D bioprinting and cartilage progenitor cells holds promise for the development of clinically translatable regenerative medicine strategies for auricular reconstruction.

## Highlights

• First application of human auricular cartilage progenitor cells for bioprinting.
• Dual-printing of hybrid ear-shaped constructs with excellent shape fidelity over time.
• Strategy and design ensured adequate deposition of cartilage-like matrix throughout large auricular constructs.

## Graphical abstract

**Figure:**
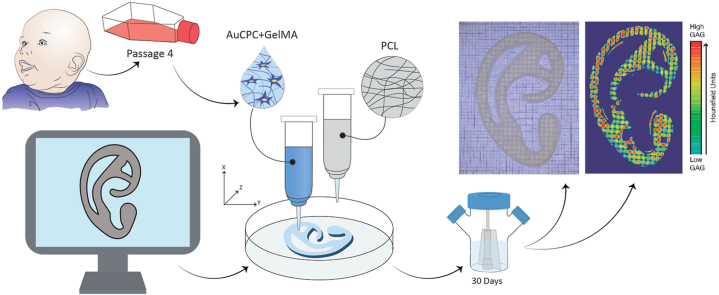

## 1 Introduction

Regenerative medicine (RM) is a promising strategy for future treatment of auricular cartilage defects and congenital malformations [[1], [2], [3]]. It typically applies a combination of cells, materials and bioactive factors to engineer a new tissue or stimulate the regeneration of native tissue [4]. As current surgical strategies for auricular reconstruction use autologous costal cartilage for shaping the implant framework [[5], [6], [7], [8], [9]], the generation of neocartilage in the laboratory would obviate the need for a large harvest site and thus reduce associated morbidity [[1], [2], [3],^10^,^11^]. In addition, RM techniques have the potential to further mimic the structural and functional complexity of native tissue [3,12]. Compared to the rigid costal cartilage framework [2,3] or the synthetic alternative porous polyethylene [13], the engineered auricular implant should ideally exhibit biochemical and mechanical properties that are more similar to the native elastic cartilage [3,14]. The first clinical trial with tissue-engineered ear-shaped constructs implanted in five children presents encouraging preliminary outcomes [15].

Nevertheless, engineered auricular constructs still face a number of challenges before they become viable alternatives for currently applied reconstructive strategies. The human auricle presents a complex structure that is difficult to fabricate and maintain. Firstly, its unique shape requires a patient-specific approach while highlighting the anatomical details to ensure an aesthetically satisfactory result after implantation under the cranial skin [16,17]. Secondly, the maintenance of that shape should be ensured for a lifetime, requiring excellent cellular performance and a long-term balance between stiffness and flexibility. This means that cartilage matrix deposition should be abundant and appropriately organized to properly mimic the native tissue's microscopic anatomy and biomechanical properties. However, especially during the first stages of tissue development and maturation, these properties are inferior to the native situation, as well as deformation and collapse are frequently reported in long-term *in vivo* studies evaluating tissue engineered ear-shaped constructs [2,18,19]. Necrosis due to nutrient limitation and an inferior mechanical integrity of the developing neo-tissue may be contributing factors to this deformation and collapse [14,16,18,20]. Third, the production of a large structure requires a significant number of autologous cells. Native chondrocytes lose their chondrogenic phenotype upon repeated expansion [[21], [22], [23], [24]] and subsequently produce a more fibrocartilage-like matrix. Mesenchymal stromal cells (MSCs) are readily expandable but may favor hypertrophic differentiation and the endochondral ossification pathway, resulting in a mineralized matrix that may lead to rigidity and implant extrusion [25,26]. These challenges taken together indicate that auricular cartilage reconstruction using RM-based approaches would benefit from customized patient-specific shapes with adequate reinforcement, potent regenerative cells and improved tissue quality before translation to daily clinical practice would be suitable. An additional challenge in the application of a tissue-engineered cartilage implant for the reconstruction of microtia is that patients have limited cranial skin available to sufficiently cover the implant [60]. High mechanical integrity of the implant is required to withstand the contractive forces of the overlying skin.

Biofabrication-based RM uses additive manufacturing technology with cells and supporting materials as building blocks to create living structures [27], with the goal to recapitulate and restore functions of native tissues [28]. Through a computer-aided design and computer-aided manufacturing (CAD/CAM) process, patient-specific and customizable shapes can be generated [12,[29], [30], [31]]. The technology's ability to deposit multiple materials with high control over the structural organization allows the fabrication of complex external and internal architectures [16]. Reinforcing scaffolds can be combined with cell-laden hydrogels to create hybrid constructs with improved performance on a biochemical and biomechanical level compared with traditional tissue engineering strategies [[32], [33], [34]]. In regard to cell type, cartilage progenitor cells hold great potential as an alternative to chondrocytes or MSCs. These cells can be harvested through a small biopsy from the patient's normal external ear or cartilage remnants on the affected side and subsequently expanded to high cell numbers while maintaining a potent chondrogenic differentiation capacity [35,36].

The way toward clinical application of engineered cartilage may well be a combination of various strategies. In view of this, our study combined smart scaffold design with a progenitor cell population for the biofabrication of an auricular cartilage structure. For the first time, we applied a population of novel human auricular cartilage progenitor cells (AuCPCs) in bioprinting with the objective to fabricate an ear-shaped construct. We evaluated cellular performance after the printing process and determined an appropriate reinforcing scaffold design to support the mechanical properties of the developing cartilage. We designed the auricular shape to match current surgical strategies to emphasize the native anatomical details and coprinted AuCPC-laden gelatin methacryloyl (gelMA) hydrogel with an ear-shaped, reinforcing polycaprolactone (PCL) framework. These dual-printed auricular constructs were cultured *in vitro* and subsequently assessed on shape fidelity and biochemical composition. The hybrid fabrication of a mechanically reinforced and anatomically emphasized structure in combination with chondrogenically potent AuCPCs provides an interesting avenue for the development of clinically translatable cartilage RM strategies.

## 2 Methods

### 2.1 Isolation of cartilage progenitor cells

AuCPCs were isolated from fresh human auricular cartilage. The auricles of recently deceased elderly donors (AuCPC-adult; *n* = 4, mean age 87.5 ± 12.3, range: 69–94 years) who had donated their bodies to science were kindly provided by the Department of Anatomy at the University Medical Center Utrecht (The Netherlands). Remnant tissue from pediatric patients undergoing otoplasty (AuCPC-pediatric; *n* = 3, mean age 7.7 ± 2.1, range: 6–10 years) and microtia reconstruction (AuCPC-microtia; *n* = 3, mean age 10 ± 3.6, range: 7–14 years) were provided by the Department of Plastic, Reconstructive & Hand Surgery at the Wilhelmina Children's Hospital (Utrecht, The Netherlands). Anonymization was performed, and tissues were obtained in accordance with the ethical guidelines of the University Medical Center Utrecht.

AuCPCs were isolated from the minced cartilage as previously described [36]. Briefly, enzymatic digestion was applied using a 0.2% pronase (Roche, USA) solution for 2 h followed by a 0.075% collagenase type II (Worthington Chemical Corporation, USA) digestion for 16 h at 37 °C. After filtration through a 100 μm cell strainer and centrifugation for 5 min at 300×*g*, pelleted cells were resuspended in Dulbecco's modified Eagle Medium (DMEM; 31966, Gibco, The Netherlands) and plated at a density of 500 cells/cm^2^ in fibronectin-coated culture flasks and incubated for 20 min at 37 °C. Non-adherent cells were removed and the remaining attached cells were cultured in chondroprogenitor expansion medium, consisting of DMEM supplemented with 10% v/v fetal bovine serum (FBS; Lonza), 1% Non-Essential Amino Acids (NEAA; Gibco), 0.2 mM l-ascorbic acid 2-phosphate (Sigma-Aldrich, The Netherlands), 100 U/mL penicillin (Life Technologies, The Netherlands), 100 μg/mL streptomycin (Life Technologies) and 5 ng/mL basic fibroblast growth factor (bFGF; Peprotech, London, UK). After expansion, cells were stored in liquid nitrogen until further use.

### 2.2 Preparation of cast and printed cell-laden hydrogel samples for assessment of cellular performance

The impact of extrusion printing on AuCPCs was assessed by performing viability, metabolic and biochemical assays. For this, AuCPCs were encapsulated in gelMA hydrogel either with or without prior extrusion.

Gelatin methacryloyl (GelMA) was synthesized according to a previously published protocol [37]. Briefly, gelatin type A (obtained from porcine skin; Bloom 175; Sigma-Aldrich) in Phosphate Buffered Saline (PBS) was functionalized with methacrylic anhydride groups to an 80% degree of functionalization. To obtain a hydrogel, a 10% w/v solution of gelMA was supplemented with 0.1% w/v 2-hydroxy-1-[4-(2-hydroxyethoxy)phenyl]-2-methyl-1-propanone (Irgacure 2959; BASF, Ludwigshafen, Germany) as a photoinitiator.

AuCPCs were expanded to passage 4 and were suspended in gelMA at a density of 1.5 × 10^7^ cells/mL. For the cast group, cylindrical constructs (diameter = 6 cm, height = 2 mm) were generated by casting the cell-laden hydrogel into a custom-made Teflon™ mold and subsequently applying UV-radiation for 5 min (wavelength λ = 365 nm, intensity E = 3 mW/cm^2^, at height of 2 cm; 144 portable UV lamp, Vilber Lourmat, Germany) to elicit free-radical polymerization. For the printed group, the cell-laden hydrogel was first extruded through a microvalve (CF300, MVC03-006; RegenHU, Switzerland) using a multimaterial bioprinting device (3DDiscovery DD 135N, RegenHU, Switzerland) at 37 °C with a pressure of 0.05 MPa and a valve opening time of 400 μs, and then resuspended and cast into the mold following the aforementioned protocol. Control samples were constructed for both groups, following the same procedures with a cell-free gelMA hydrogel.

Samples were cultured in static conditions at 37 °C and 5% CO_2_ in chondroprogenitor differentiation medium, consisting of DMEM supplemented with 1% v/v insulin-transferrin-selenous acid (ITS+ Premix; Corning, USA), 0.2 mM l-ascorbic acid 2-phosphate (Sigma-Aldrich), 100 U/mL penicillin (Life Technologies), 100 μg/mL streptomycin (Life Technologies), 100 nM dexamethasone (Sigma-Aldrich) and 10 ng/mL transforming growth factor β1 (TGF-β1; Peprotech). Culture medium was refreshed every 3 days.

### 2.3 Assessment of cell viability of AuCPCs in cast and printed samples

The effect of extrusion printing on the viability of AuCPCs was assessed using a LIVE/DEAD cell viability assay. On days 1, 3, and 10 of culture, cylindrical hydrogel samples from the cast and printed group (16–19 replicates per condition) were cut in half and were subsequently incubated in 0.1% calcein-AM and 0.1% ethidium homodimer-1 (Live Technologies, USA) for 20 min on a shaker plate at room temperature. Cells were visualized using a confocal microscope (Leica SP8 X, USA), displaying a green and red color for live and dead cells, respectively. Each sample was assessed from the cut edge, revealing the center of the constructs. Per sample, 3 images were taken randomly in the *x* and *y* direction. Live and dead cells were counted and averaged, and cell viability was calculated according to the following formula:

$$Cell\;viability=\frac{live\;cells}{\;live+dead\;cells}*100\%$$

### 2.4 Evaluation of metabolic activity of AuCPCs in cast and printed samples

Metabolic activity as an indicator of cellular health was evaluated using a resazurin assay on days 1, 3, and 10. A 440 mM stock solution of resazurin (Alfa Aesar, Germany) was diluted with differentiation medium in a 1:10 ratio. Subsequently, hydrogel samples from the cast and printed group (8 donors, 3 replicates per donor) were incubated in this solution in the dark at 37 °C. After 4 h incubation, fluorescence of resorufin (reduced from the resazurin agent) was measured at 544 nm excitation and 570 nm emission using a spectrofluorometer (Fluoroskan Ascent FL; ThermoFisher, USA). The resulting fluorescence is reported here, after correction for blanks.

### 2.5 Quantification of glycosaminoglycan production in cast and printed samples

At day 28 of culture, 16 replicates of each group were collected and prepared for biochemical evaluation. Lyophilized samples were digested overnight at 60 °C in 250 μL papain digestion buffer (P3125; Sigma-Aldrich), consisting of 0.2 M NaH_2_PO_4_ (Merck, USA) and 0.01 M ethylenediaminetetraacetic acid (VWR, USA) in milliQ water (pH = 6.0), supplemented with 250 μL/mL papain solution (48 units/mg of protein; Sigma-Aldrich) and 0.01 M cysteine (C9768; Sigma-Aldrich).

Total double-stranded DNA (dsDNA) content was quantified using a Quant-iT PicoGreen dsDNA assay (Life Technologies). A spectrofluorometer (Fluoroskan Ascent FL; ThermoFisher) was used to measure fluorescence at 485 nm excitation and 520 nm emission. Results were corrected for the dilution factor and compared to a standard of known concentrations of DNA.

Glycosaminoglycans were quantified as a measure of cartilage-specific matrix production following a demethylmethyleneblue (DMMB; Sigma-Aldrich; pH = 3.0) assay. The 525/595 nm absorbance ratio of the reagent was determined with a VersaMax plate reader (Molecular Devices, UK). Taken into account the dilution factor, a standard of known concentrations of chondroitin sulfate C was used to calculate the content of sulfated gycosaminoglycans (GAG).

Sulfated GAG (sGAG) and dsDNA content in each sample were both normalized against the dry weight of the sample. The ratio of sGAG per dsDNA was calculated to display the cartilage-specific matrix-production activity of single cells in the hydrogel.

### 2.6 Preparation of supporting PCL scaffolds with various strand spacings

Properties of supporting scaffolds were evaluated through mechanical and biochemical assessment. For this, printed scaffolds with various fiber spacings were fabricated and combined with cell-free or cell-laden hydrogel.

Supporting scaffolds were printed through fused deposition modeling (FDM) using the 3D Discovery bioprinter. Fabrication occurred in a layer-by-layer manner to create a woodlog 0°–90° organization with strand spacings of 400 μm, 800 μm, 1000 μm, and 1200 μm. Medical grade poly-ε-caprolactone (PCL; Purasorb PC 12; Corbion Inc., The Netherlands) was heated to 80 °C in the printhead and extruded through a 27 G needle at a pressure of 0.6 MPa with a feed rate of 0.7 mm/s, resulting in a strand thickness of 300 μm. Scaffolds were first printed in 60 mm (L) x 10 mm (W) x 2 mm (H) sheets, after which cylindrical samples were obtained by applying a biopsy punch (diameter = 5 mm; BAP Medical, The Netherlands).

### 2.7 Compression testing on supporting scaffolds

To evaluate compressive properties of printed PCL scaffolds of the varying strand spacings, both PCL scaffold-only and hybrid hydrogel-PCL samples (*n* = 5) were subjected to unconfined uniaxial compression testing. For the hybrid group, scaffolds were inserted into the previously described Teflon™ mold and injected with a photoinitiator-supplemented 10% w/v gelMA hydrogel, followed by UV-crosslinking as described previously. Hybrid samples were submerged in PBS for 24 h before testing. Tests were performed on a static mechanical testing device (Zwick Z010; Zwick Roell Kennesaw, USA) with a 1 kN loadcell. Samples were subjected to a compressive load at a constant crosshead speed of 1 mm/min. The compressive modulus was obtained from the slope of the engineered stress/strain curves at in the 10–15% strain. The unloaded cross-sectional of the cylindrical samples was used to determine the engineered stress.

### 2.8 Biochemical evaluation of cell-laden hybrid scaffolds

Hybrid scaffolds were prepared in the same manner as described previously, yet with a 10% w/v hydrogel laden with AuCPCs at a density 1.5 × 10^7^ cells/mL. Cell-laden gels without supporting PCL scaffolds were fabricated as controls. Samples were cultured in chondroprogenitor differentiation medium at 37 °C and 5% CO_2_. Medium was refreshed 3 times per week. After 28 days of culture, samples were harvested and prepared for biochemical testing. Quantification of GAGs and DNA was performed as previously described.

### 2.9 Hybrid bioprinting of ear-shaped constructs and chondrogenic differentiation in dynamic culture

As a proof-of-principle, auricular constructs were fabricated through coprinting of human AuCPCs with a reinforcing PCL scaffold. After 30 days of *in vitro* culture, the printed ears were assessed on shape fidelity and biochemical composition by means of microcomputed tomography scanner (μCT) scanning and processing, biochemical quantification, histology, and immunohistochemistry.

A modular auricular implant was designed that aligned with current surgical strategy and resulted in a satisfactory esthetic appearance, as reported previously [16]. The base module of this design was used for this proof-of-principle experiment, with dimensions of 38.9 (L) x 25.35 (W) x 2.0 (H) mm. The 3D ear model was transcribed into the corresponding G code for the 3DDiscovery bioprinter. Using the previously reported printing parameters, contoured PCL scaffolds were printed with a strand spacing of 1000 μm.

AuCPCs of one pediatric donor were expanded up to passage 4 and encapsulated in gelMA at a density of 1.5 × 10^7^ cells/mL. The cell-laden hydrogel was extruded through a microvalve into the ear-shaped scaffold. Each scaffold required 1 mL of cell-laden hydrogel. The hybrid constructs were immediately photocrosslinked by UV-radiation for 15 min (wavelength λ = 365 nm, intensity E = 7 mW/cm^2^, at a height of 12 cm; CL-1000L UV Crosslinker, UVP, UK).

Printed ear-shaped constructs were cultured at 37 °C and 5% CO_2_ in chondroprogenitor differentiation medium for 30 days in a Spinner flask bioreactor with a stirring rate of 18 rpm. Medium was refreshed twice per week.

### 2.10 Assessment of distribution of glycosaminoglycans through contrast-enhanced microcomputed tomography and histology

To assess the overall geometry of the printed ear-shaped constructs, as well as the 3D distribution of neo-synthesized cartilage matrix, samples were harvested at day 1 and day 30 (*n* = 3) and incubated in a PBS solution containing 12 mg/mL of CA4+ (MW = 1354 g/mol, q = +4) for 4 h at 37 °C. The cationic contrast agent CA4+, exhibiting high affinity for negatively charged glycosaminoglycans, was synthesized as previously described [38]. After incubation, the samples were removed from the contrast agent solution and scanned with a μCT (Quantum FX, Perkin Elmer, USA). For X-ray attenuation measurements, this occurred at a voxel size of 60 μm^3^ with 70 kV tube voltage and 200 μA tube current for 17 s. For comparison with histology, voxel size was 50 μm^3^, tube voltage was 70 kV, and tube current was 200 μA for 4.30 min 3D reconstruction was carried out automatically after completion of each scan using the scanner's software (Quantum FX μCT software, Perkin Elmer, USA). Image analysis was performed using Fiji (software version 1.50; National Institutes of Health, USA) [39]. Mean X-ray attenuation values on the Hounsfield scale were obtained by averaging attenuation values over all the coronal μCT slides.

Constructs for histology were fixed in 4% neutral-buffered formalin, dehydrated through a graded ethanol series, cleared in xylene and embedded in paraffin. Constructs were sectioned into slices of 5 μm thickness in the same direction and orientation as the μCT slices. The deposition of cartilage glycosaminoglycans was evaluated through a triple stain consisting of hematoxylin, fast green and safranin O. Collagens were visualized through immunohistochemistry as previously reported [16], with appropriate antibodies for collagen type II (II-II6B3; DSHB, USA) and collagen type I (ab138492, 1:400; Abcam, UK). All sections were mounted in DPX mounting medium (Millipore, USA) and examined using a light microscope (Olympus BX51; Olympus, Germany). Histological images were compared with their corresponding slices in the μCT stacks using Fiji.

### 2.11 Determination of shape and size retainment of printed ear-shaped constructs

Retainment of shape of the printed ear-shaped constructs was evaluated by comparing the 3D images obtained by μCT at day 1 and day 30 to the original digital design. The μCT images (pixel dimensions 0.118 × 0.118 × 0.118 mm) were segmented and converted to a dense 3D surface mesh model using 3D Slicer [40]. Incomplete hydrogel filling of the scaffold was corrected by manual closure of the holes to accommodate a clean comparison of scaffold shapes. Shape comparison requires dense models with evenly distributed 3D points; hence, all models including the original digital design were remeshed in Meshmixer (Autodesk, USA). Subsequently, the printed ear models were compared with the original design. First, both models were aligned in the coordinate system through the Iteratively Closest Point (ICP) algorithm using the alignment tool in MeshLab (Visual Computing Lab, Italy). Then, the distance of each 3D point in the printed ear model to its closest corresponding 3D point in the original design model was calculated using the Hausdorff distance filter in MeshLab. The minimal maximal, mean, and root mean square (RMS) distance of each comparison were determined and the distances were visualized in a 3D color map. A margin of 1.5 mm was determined as an acceptable maximum deviation from the original digital design [15].

### 2.12 Statistical analysis

Quantitative results are expressed as mean ± SEM. Comparisons between cast and printed cells at different time points were performed through a two-way ANOVA. Results of mechanical properties of scaffolds were assessed using a two-way ANOVA, whereas biochemical composition between the various strand spacings was evaluated using an ordinary one-way ANOVA. Bonferroni post-hoc tests were applied to these analyses. Quantitative results from the printed ears were analyzed with an unpaired *t*-test. Statistical analyses were carried out using Graphpad Prism 8 (Graphpad Software, USA). A value of *p* < 0.05 was considered statistically significant.

## 3 Results

### 3.1 Extrusion printing does not negatively affect cell viability, metabolic activity, and GAG production

**Fig. 1:**
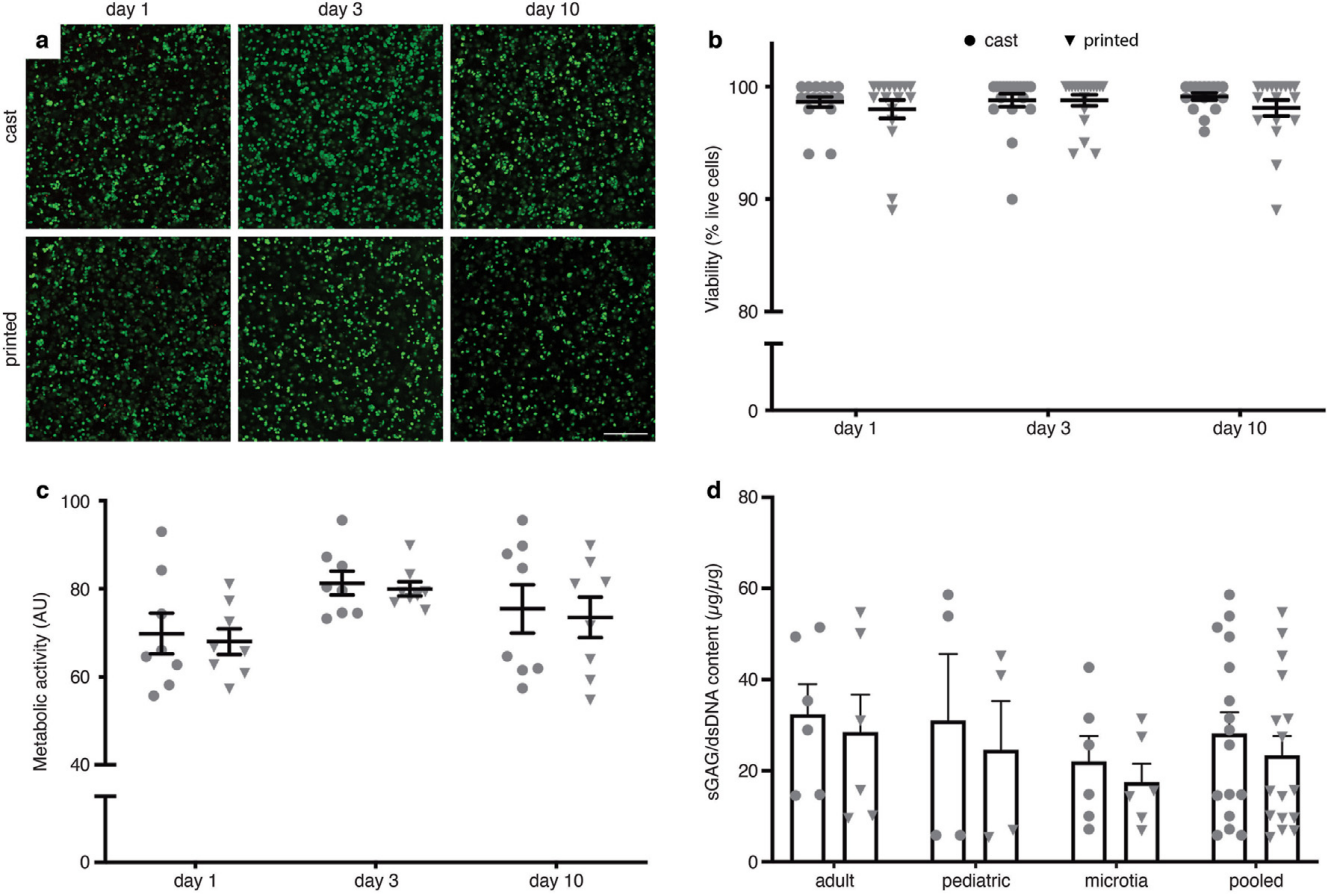
*Effects of extrusion printing on viability, metabolic activity, and chondrogenic differentiation of AuCPCs.* (a) LIVE/DEAD staining on cast and printed constructs after 1, 3, and 10 days of culture. Representative images of one pediatric donor are shown, where green indicates live cells and red indicates dead cells. Scale bar equals 250 μm. (b) Quantification of live cells as a percentage of the total counted cells at the different time points. (c) Fluorescence of resorufin as an indication of metabolic activity at days 1, 3, and 10, expressed in means per donor. (d) Production of cartilage-specific proteoglycans by quantification of glycosaminoglycans after 28 days in chondrogenic culture. Replicate outcomes are reported in panels b/c/d, as well as the mean. Error bars represent SEM. No significant differences (p < 0.05) between cast and printed conditions were observed.

LIVE/DEAD staining was performed on AuCPCs in gelMA hydrogel at days 1, 3, and 10. Both cast and printed groups displayed predominantly green stained cells throughout the samples at all time points (Fig. 1a). Cells were distributed homogeneously throughout the gel. Quantification of live and dead cells confirmed high viability rates in both cast and printed groups (Fig. 1b). Cast constructs displayed 98.62% ± 0.43, 98.82% ± 0.56, and 99.12% ± 0.3 live cells at day 1, day 3, and day 10, respectively. Cells within printed constructs performed similarly, with a viability rate of 97.98% ± 0.81, 98.72% ± 0.52, and 98.10% ± 0.73 at days 1, 3 and 10, respectively. No significant differences were observed between cast and printed groups and over time.

Metabolic activity as measured through fluorescence of resorufin (Fig. 1c) was 69.85 ± 4.61, 81.29 ± 2.72, and 75.45 ± 5.45 arbitrary units (AU) at days 1, 3, and 10 in the cast group. Values of printed constructs were comparable: 68.04 ± 2.91, 79.99 ± 1.63, and 73.53 ± 4.62 at days 1, 3, and 10, and no significant differences were observed between groups.

Production of GAGs was assessed after 28 days of *in vitro* chondrogenic culture (Fig. 1d). No significant differences in GAG production were observed between groups. The mean GAG per DNA of cast samples was 28.17 ± 4.65 μg/μg and for printed samples this was 23.41 ± 4.20 μg/μg. Nevertheless, there were notable differences between individual donors in each separate donor group (adult, pediatric and microtia), where for some donors only little matrix synthesis was observed. AuCPCs from adult donors produced a mean GAG per DNA of 32.42 ± 6.59 μg/μg when cast and 28.51 ± 8.21 μg/μg when printed. Cells from pediatric auricular cartilage produced 31.05 ± 14.60 μg/μg in cast constructs, whereas cells in printed samples produced 24.60 ± 10.68 μg/μg. Constructs with microtia-derived AuCPCs contained 22.00 ± 5.62 μg/μg GAG per DNA when cast and 23.41 ± 4.20 μg/μg when printed.

### 3.2 PCL scaffolds with various strand spacings allow for glycosaminoglycan production

**Fig. 2:**
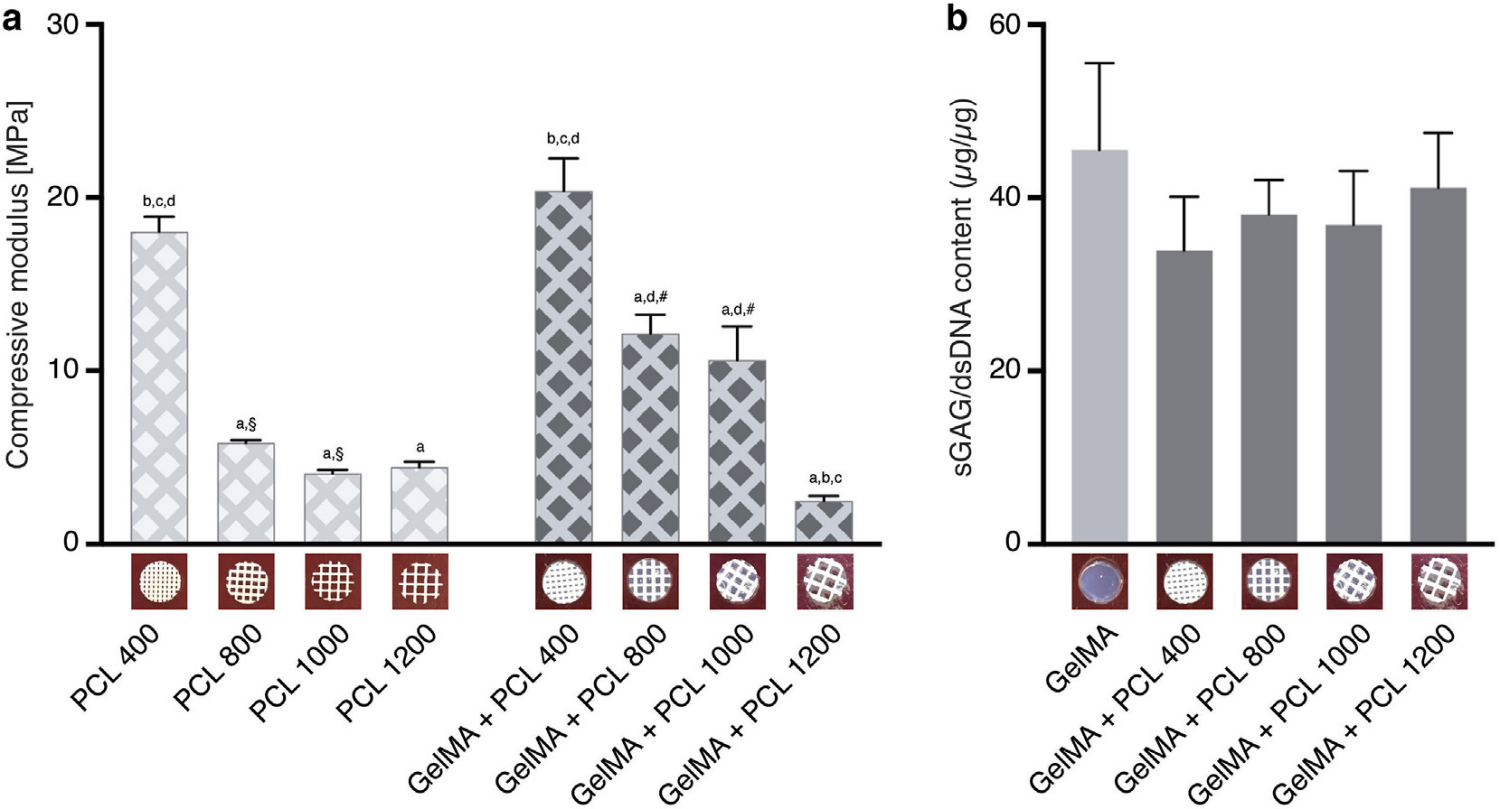
*Scaffolds with varying strand spacings exhibit different compressive properties but display similar GAG production.* (a) Compressive modulus of PCL scaffolds and hybrid GelMA + PCL scaffolds with strand spacings of 400, 800, 1000, and 1200 μm. (b) Production of glycosaminoglycans in hydrogel and hybrid GelMA + PCL samples, as normalized to dsDNA content. Error bars represent SEM. A significant difference in compressive modulus to the 400 μm condition is indicated by *a*, to the 800 μm condition by *b*, to the 1000 μm condition by *c,* and to the 1200 μm condition by *d*. § indicates a significant difference from the PCL-only condition to the hybrid condition, and # signifies a significant difference from the hybrid condition to the PCL-only condition. No significant differences in glycosaminoglycan production were observed. PCL, polycaprolactone.

FDM-printed PCL scaffolds were fabricated with strand spacings of 400 μm, 800 μm, 1000 μm, and 1200 μm. Compressive properties of empty scaffolds and hybrid scaffolds with 10% gelMA were determined (Fig. 2a). Scaffolds of 400 μm without gel exhibited a compressive modulus of 18.0 ± 0.9 MPa; significantly higher than 5.9 ± 0.1 MPa, 4.1 ± 0.2 MPa, and 4.4 ± 0.3 MPa in 800 μm, 1000 μm, and 1200 μm scaffolds, respectively. Incorporating 10% w/v gelMA into the 800 μm and 1000 μm scaffolds significantly increased the compressive modulus to 12.2 ± 1.1 MPa and 10.6 ± 1.9 MPa, respectively. The effect of incorporating reinforcing fiber structures on the production of GAGs was assessed by comparing hybrid constructs with non-reinforced, hydrogel-only samples (Fig. 2b). No significant effect of incorporating PCL fibers was observed on the ability of AuCPCs to produce GAGs for a period of 28 days.

**Fig. 3:**
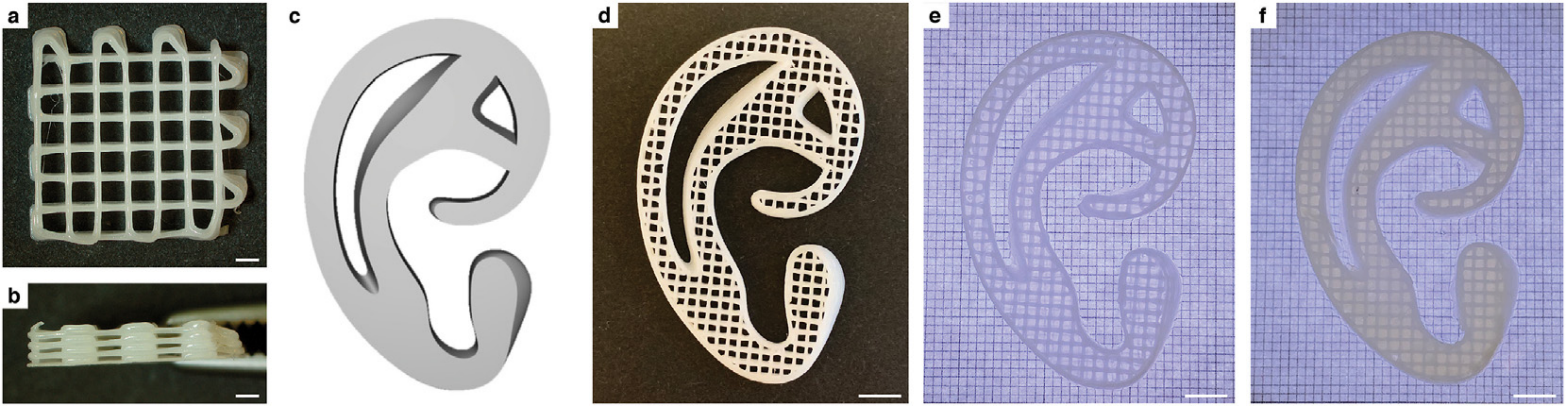
*Fabrication of the PCL-based reinforcing auricular scaffolds.* (a) Top and (b) side view of scaffolds with 1000 μm strand spacing. (c) Digital 3D model of auricular module. (d) PCL scaffold of the auricular module. (e) Hybrid cell-laden gelMA-PCL auricular modules directly after printing, and (f) after 30 days of *in vitro* culture, imaged by stereomicroscope. Scale bars equal 1000 μm in panels a/b and 5 mm in panels d/e/f. PCL, polycaprolactone; gelMA, gelatin methacryloyl.

Straight non-sagging fibers could be produced using the FDM printing technique. Fabricated scaffolds demonstrated excellent top (Fig. 3a) and side (Fig. 3b) porosity. The digital 3D model of the auricular structure (Fig. 3c) could reliably be fabricated with a strand spacing of 1000 μm while maintaining fiber quality and porosity (Fig. 3d). Cell-laden gelMA hydrogel was homogenously distributed throughout the auricular scaffold (Fig. 3e), with a few local exceptions where gel had leaked out after printing. During 30 days of dynamic *in vitro* culture, the hybrid auricular structures remained intact (Fig. 3f).

### 3.3 Excellent shape fidelity of printed constructs directly after printing and after 30 days of *in vitro* culture

**Fig. 4:**
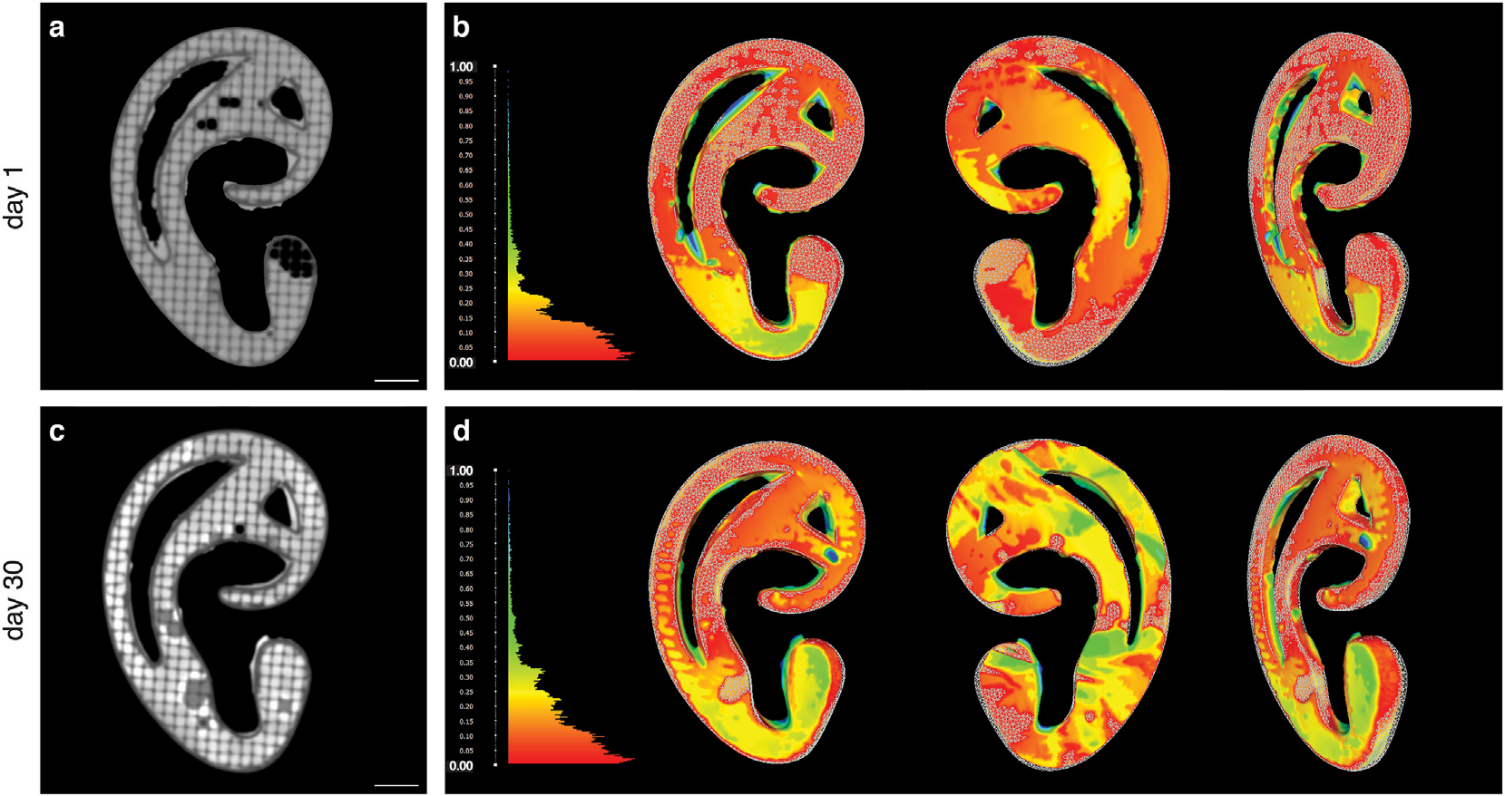
*Assessment of shape fidelity of printed ear-shaped constructs in 3D*. μCT images of ear-shaped constructs in coronal plane after 1 (a) and 30 (c) days of *in vitro* pre-culture. Scale bars in panels a/c equal 5 mm. Shape conformity of printed shapes compared to the original digital design after dynamic culture of 1 day (b) and 30 days (d). The Hausdorff distances from each point in the printed shape to the closest point in the digital model are visualized in a color distance map, ranging from red (0.00 mm) to blue (1.00 mm). The digital design is visualized as a triangle mesh.

Shape accuracy after printing and retainment in *in vitro* culture was determined through μCT scanning (Fig. 4a and c) and subsequent computing of the distance between closest corresponding 3D points (Fig. 4b and d). The day after printing, the fabricated auricular shape corresponded to the original digital design with a mean deformation of 0.13 mm (min = 0 mm, max = 1.80 mm, RMS = 0.19 mm). After 30 days in culture, average deviation from the digital model increased to 0.21 mm (min = 0 mm, max = 1.47 mm, RMS = 0.28 mm). This increase of 0.08 mm is less than the spatial resolution of the μCT (voxel size = 0.118). Both distance color maps display an array of predominantly red, orange, and yellow colors, indicating distances of <0.5 mm.

### 3.4 Cartilage-specific components are produced abundantly throughout the hybrid ear-shaped construct

**Fig. 5:**
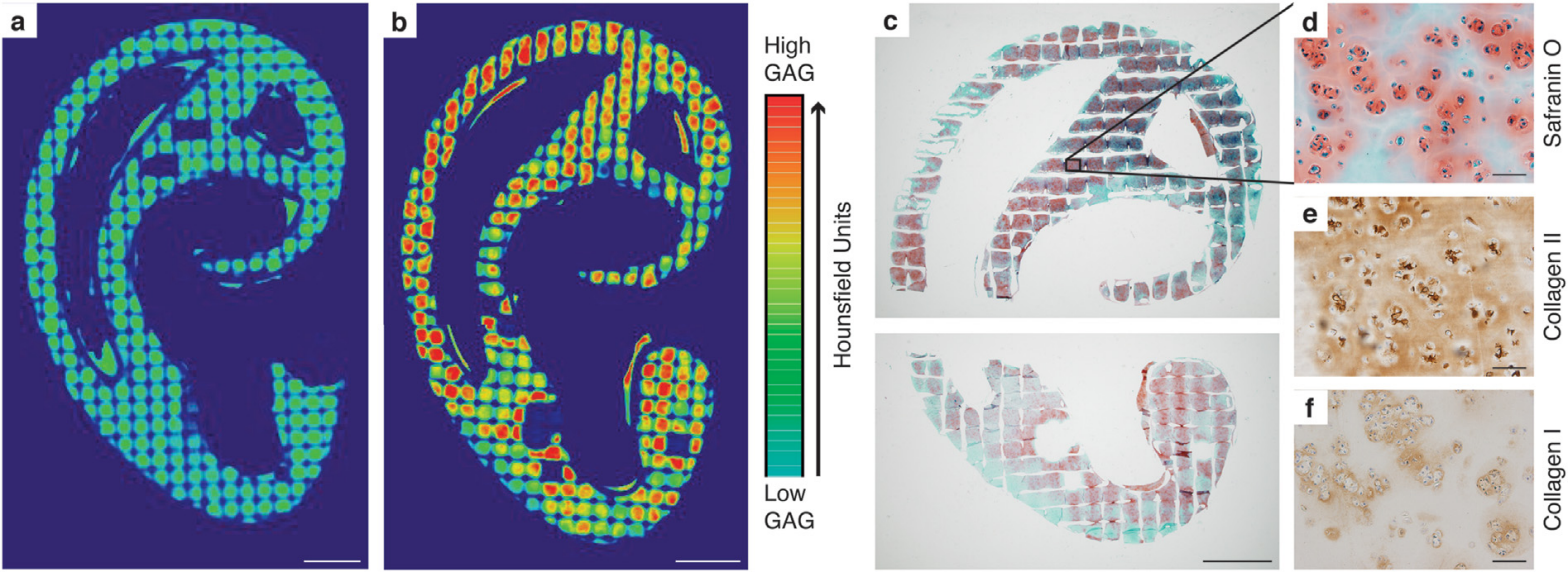
*Qualitative analysis of deposition of cartilage matrix components in bioprinted auricular constructs with pediatric AuCPCs after 30 days of in vitro culture.* X-ray attenuation obtained by μCT scanning after CA4+ incubation at day 1 (a) and day 30 (b), as expressed in Hounsfield Units. Distribution of glycosaminoglycans throughout the auricular sample (c) and at 10× magnification (d). Deposition of collagen type II (e) and collagen type I (f). Scale bars in panels a/b/c equal 5 mm and in panels d/e/f equal 100 μm. μCT, microcomputed tomography scanner.

**Fig. 6:**
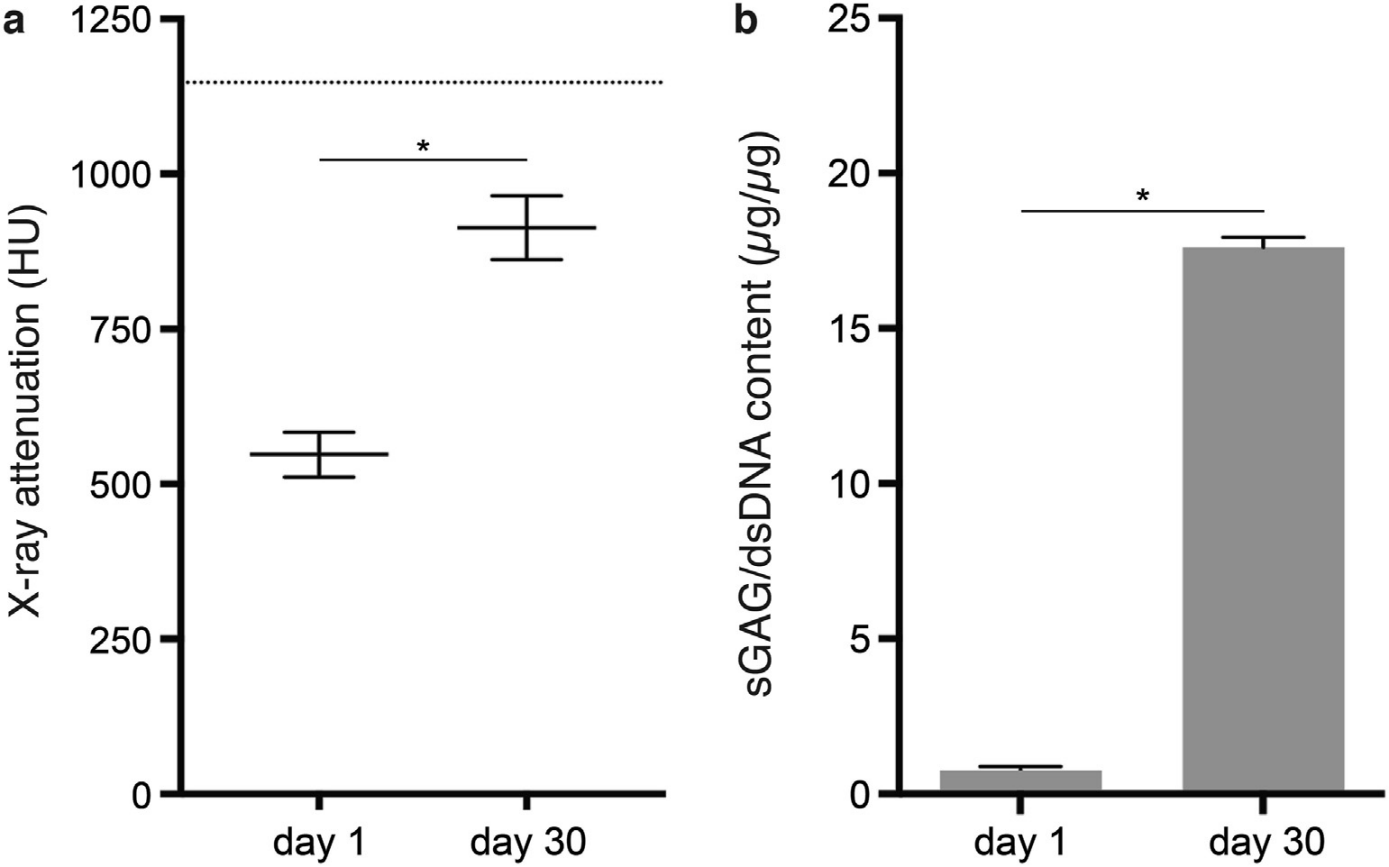
*Quantitative analysis of glycosaminoglycan production of bioprinted auricular constructs with pediatric AuCPCs.* (a) Mean X-ray attenuation values on the Hounsfield scale and (b) mean GAG per DNA after 30 days of *in vitro* culture. The mean value from a sample of native pediatric auricular cartilage (1 donor, 2 replicates; 1147.6 HU) is included as a reference (dashed line). Error bars represent SEM. The asterisk (∗) indicates a significant difference (p < 0.05) between time points.

The distribution of GAGs within printed cell-laden ear-shaped constructs was determined by contrast-enhanced μCT and histological evaluation. Compared with day 1 (Fig. 5a), samples cultured for 30 days displayed increased GAG content, as measured by an increase in X-ray attenuation (Fig. 5b). X-ray attenuation values increased significantly over the 30-day culture period, with a mean of 547.7 ± 35.9 HU at day 1 and 913.2 ± 51.4 HU at day 30 (Fig. 6a). GAGs were distributed throughout the auricular module, with qualitatively higher intensities in the helix, antihelix and tragus areas (Supplementary Video 1). Production and distribution of GAGs was confirmed through Safranin O staining of a corresponding slice (Fig. 5c). There was abundant pericellular labeling gradually dispersing into the inter-territorial matrices (Fig. 5d). GAG production was confirmed through biochemical analysis, which showed a significant increase compared to day 1 (Fig. 6b). Mean GAG per DNA was 17.62 ± 0.32 μg/μg at day 30 compared with 0.74 ± 0.16 μg/μg at day 1. Collagen type II displayed a similar organization as glycosaminoglycans, with intense intracellular brown staining with a gradual distribution into a wide interterritorial organization (Fig. 5e). Collagen type I staining was of less intensity and localized mainly in the pericellular areas (Fig. 5f).

Supplementary video related to this article can be found at https://doi.org/10.1016/j.mtbio.2021.100094

The following is/are the supplementary data related to this article:

Supplementary Video 1 *Z-stack representation of glycosaminoglycan distribution in auricular constructs*. Glycosaminoglycan distribution in each segment of the auricular construct after 30 days of in vitro culture, as obtained by uCT scanning after CA4+ incubation. Supplementary Video 1

## 4 Discussion

RM-based treatment of auricular deformities offers the potential to surpass clinical outcomes of the current gold standard reconstructive techniques. Nevertheless, the intricate three-dimensional shape of the auricle, the biochemical composition of the auricular cartilage, and its biomechanical properties present ongoing challenges in the fabrication of auricular implants. Several promising strategies have been reported in terms of patient-specific implant design [29,31,[41], [42], [43], [44]], cartilage-like tissue regeneration [36,[45], [46], [47]], construct reinforcement [48,49] and hybrid bioprinting [30,50,51]. Digital photogrammetry and CAD/CAM technology can aid in the creation of patient-specific auricular shapes [29,31,44,52,53] with improved internal spatial organization. Kim et al. 3D printed porous polyurethane auricular scaffolds that demonstrated improved cartilage ingrowth in comparison to commercially available MedPor® implants [54]. In the study by Zopf et al. [12], 3D-printed patient-specific auricular scaffolds seeded with chondrocytes were implanted subcutaneously in mice and showed evidence of shape maintenance and neocartilage matrix deposition *in vivo*. Kang et al. [30] presented an integrated tissue-organ printer with which reinforced hybrid auricular constructs were fabricated that displayed cartilage matrix production after 2 months *in vivo*. Tissue-organ printing systems have also been successful in the fabrication of multitissue auricular constructs, as exemplified by the studies by Lee et al. [55] and Jung et al. [51] where both the auricular cartilage and the fatty earlobe were printed in a composite construct. Despite these collective efforts, the ‘bioengineered auricle’ is not yet ready for widespread clinical application. Indeed, only one study has thus far described the application of tissue-engineered auricular constructs in patients, with encouraging results. Zhou et al. 3D printed a patient-specific mold in which autologous microtia chondrocytes were seeded onto a composite polymer scaffold and implanted in 5 children after a 12-week *in vitro* preculture period. Evident cartilage formation was observed before implantation, and there were no signs of absorption or extrusion during a 2.5-year follow-up [15]. Nevertheless, the histological and esthetical outcomes do not fully match the composition and complex geometry of the native auricle yet. Patient-specific shapes, adequate construct reinforcement, enhanced cellular function and improved quality of the neo-tissue remain key challenges for clinical translation of engineered auricular structures. Future RM-based approaches for auricular cartilage reconstruction may benefit from a smart combination of various strategies and technologies.

Our approach entails fabricating a hybrid ear-shaped scaffold using bioprinting techniques, a recently identified progenitor cell population, previously validated biomaterials and smart scaffold design. Microvalve printing proves superior to casting with regards to spatial organization and homogenous cell seeding [89]. Combined gelMA and PCL hybrid constructs are suitable for RM purposes, as evidenced by successful outcomes in terms of printability, construct stability, and tissue regeneration in various studies [34,49,56]. The hydrogel gelMA supports chondrogenic differentiation of cartilage progenitor cells [36,56], is suitable as a bioink [57] and is available in clinical-grade quality [57]. Although the gelatin type A used in this hydrogel harbors endogenous endotoxins that may elicit an inflammatory response in more complex *in vitro* or *in vivo* environments [57], a recent study determined no critical effects of endotoxin level on chondrogenic differentiation [88]. The physicochemical properties of PCL enable additive manufacturing of porous 3D scaffolds that support construct integrity as well as neotissue formation [49,58,59]. Results from our study underscore that the incorporation of fiber reinforcement does not affect the ability of embedded cells to produce matrix, as exemplified by the non-significant differences in glycosaminoglycan production in samples with strand distances between 400 and 1200 μm. Furthermore, these reinforcing scaffolds enable a necessary increase in compressive properties of hydrogel constructs. In previous studies, we found a maximum compressive modulus of cell-laden gelMA hydrogels of 179.2 kPa after 56 days of *in vitro* culture [36]. Although a significant increase over time was observed compared with the start of culture, these values are insufficient to support the developing neotissue after implantation *in vivo*, where contractive forces of the overlying tissues, as well as external forces impact the implanted engineered construct [60,61]. Reports on mechanical characterization of the native auricular cartilage in humans are limited, and test types and outcome measures vary between studies [[62], [63], [64]]. Griffin et al. [63] reported the compressive Young's modulus of native human auricular cartilage to range between 1.41 MPa in the helix and 2.08 MPa in the concha of the ear. Clearly, tissue-engineered auricular constructs require structural enhancement to sustain the mechanical loading that the construct may experience *in vivo*. This challenge is recognized and various approaches are described in the literature to accomplish mechanical reinforcement. Support of the developing neotissue has initially been provided through external stents [[65], [66], [67]] or molds [68]. Visscher et al. (2019) described a 3D-printed internal porous PCL mold to encapsulate a cell-laden hydrogel that exhibited compressive moduli between 100 and 200 MPa depending on porosity [69]. Although this approach will certainly protect the developing neotissue within, this high degree of construct stiffness may cause similar problems to porous polyethylene, including extrusion and exposure [70]. The incorporation of a metal wire framework in the study by Zhou et al. maintained the dimensions of ear-shaped constructs *in vivo* [71], yet this approach also harbors a high risk of implant extrusion and exposure. The work by Melchels et al. presents a more subtle hydrogel-based reinforcement strategy that enabled tuning of the composite material stiffness between 138 and 263 kPa [48]. A hybrid auricular construct was printed where the cell-laden bioink was reinforced with a strengthening hydrogel. This approach provides a highly cell-friendly environment, yet the compressive properties may still be unable to support the developing neocartilage upon implantation. Fiber reinforcement using biodegradable polymers can further increase biomechanical characteristics of engineered cartilage. PCL has previously been used to fabricate various complex structures [34] and markedly increases construct stiffness when combined with a hydrogel [49]. The studies by Zhou et al., Kang et al., Park et al., Jung et al., Zopf et al. and Lee et al. have all demonstrated the successful 3D fabrication of PCL-reinforced auricular constructs [12,15,30,32,51,55]. In our study, compression tests on cylindrical constructs were performed to determine appropriate strand spacing for the supporting PCL scaffold. We found that reinforcing gelMA samples with FDM-printed PCL fibers increases the compressive modulus to at least 2.49 MPa. This increase in modulus indicates that such fiber-reinforcement is sufficient to potentially support the engineered auricular construct during tissue maturation *in vivo*, without causing unnecessary rigidity. In addition, the modulation of scaffold porosity can adjust the compressive modulus [90] in specific areas of the construct to mimic the spatiotemporal variation in mechanical properties in the native human ear [62].

A limitation of the current auricular reconstruction strategies is that the implanted material, which is either autologous costal cartilage or synthetic porous polyethylene, is quite stiff and has an unnatural feel [3]. The human auricle is a flexible structure that endures many daily stresses, such as wearing glasses, headphones or a helmet, the rubbing of clothes while dressing, and sleeping or leaning on it. Rigid structures may cause discomfort and pain, and potentially also soft tissue inflammation, skin necrosis, and implant exposure or extrusion even after light traumas [3,72,73]. The risk of incorporating a stiff fiber network with a high compressive modulus in the tissue-engineered construct is that the structure can become too rigid. Therefore, it is important to create reinforcement that provides both stiffness as well as flexibility. Upon handling the scaffolds in this study, the 400 μm and 800 μm scaffolds are quite stiff and inflexible, whereas the 1000 μm and 1200 μm scaffolds allowed an increasing degree of bending upon manual manipulation. However, with increasing strand spacing also comes less control over the fine architecture of the structure. For our proof-of-concept study, we selected the pattern that provided properties closest to the overall native situation without compromising on the structural integrity of the ear-shaped construct. Progress in bioprinting technology could offer new possibilities for improved internal organization. For instance, an interesting option would be to incorporate organized microfibrous 3D PCL meshes fabricated through the melt electrowriting technique into hybrid constructs. These fibers markedly increase the compressive and shear properties of hydrogel-thermoplastic constructs [49,[74], [75], [76]] and may allow improved flexibility of engineered auricular constructs without compromising on other key features. Although this was not tested in our study, it would be advisable for future studies to include 3-point bending tests to assess mechanical characteristics of tissue-engineered auricular constructs [77]. Information on bending behavior of scaffolds would help in designing reinforcing structures with more refined mechanical attributes that can mimic the native situation.

Proper reinforcement of the engineered auricle offers initial mechanical stability and thus protects implant shape, yet there are also other factors at play impeding the success of the auricular implant. Tissue maturation may be hampered by a limited nutrient supply in avascular constructs [16]. Especially central regions in large constructs may receive too little nutrients for proliferating and differentiating cells to flourish, leading to necrosis and construct deformation [20,78]. We previously proposed a modular approach in which the full auricular implant is made up of separately fabricated and matured parts that are combined in a later stage [16]. In addition, the design of the auricular shape is based on the current surgical strategy with an open framework, omitting areas in the scapha, fossa triangularis and concha, and emphasizing the natural eminences and depressions of the auricle to take into account the thickness of the overlying skin. This way, the anatomical details are preserved when the auricular implant is covered with skin or facial flaps. More importantly, the construct's surface area for diffusion is maximized and diffusion distances for oxygen and other essential nutrients are decreased when compared with full-thickness auricular constructs. Previous experiments conducted by our group have demonstrated that articular cartilage progenitor cells maintain metabolic activity in larger 3D shapes over the course of 28 days [87]. In our study, qualitative analysis of the distribution of matrix components demonstrated glycosaminoglycan deposition throughout the auricular module, including central areas. Nevertheless, this distribution was non-homogenous and not all areas displayed optimal matrix production after 30 days *in vitro*. An additional strategy, although non-reflective of the native situation, would be to create perfusion channels to allow non-obstructed flow of nutrients into the construct. Kang et al. reported improved cartilaginous matrix formation throughout auricular constructs due to the incorporation of microchannels [30]. Another interesting strategy would be to provide a reservoir of nutrients within the engineered constructs to alleviate metabolic stresses during periods of high nutrient requirement. Armstrong et al. [79] delivered additional oxygen to cells in central areas of large constructs through myoglobin complexes on the cell membrane. Innovative approaches like these can tremendously improve cell survival and tissue development in large engineered constructs similar to the auricle.

The quality of the neotissue is not only impacted by the nutrient supply but importantly also by the inherent regenerative potential of the embedded cells. The fabrication of the human auricle requires between 100 and 250 million chondrogenically potent cells [2,18] that should be able to generate an organized neotissue that is rich in glycosaminoglycans, collagens, and elastin. Hence, cell choice is a crucial factor in the success of the engineered implant, and options that have extensively been explored for cartilage tissue engineering include chondrocytes and MSCs from various sources. Native chondrocytes are dedicated to chondrogenesis but are also limited by a dedifferentiation process after repeated expansion, resulting in an inferior fibrocartilage-like matrix [2,22,23,[80], [81], [82], [83]]. MSCs, on the other hand, are readily expandable while maintaining multipotency; however, these cells display a tendency to undergo hypertrophy and differentiate toward the osteogenic lineage [25,26]. A recent addition to these choices is the subpopulation of progenitor cells residing in the auricular cartilage [36,84]. These cells can be obtained through a non-deforming biopsy from the auricle and can be expanded to high cell numbers while maintaining a chondrogenic phenotype, and they are able to produce a cartilage-like matrix in gelMA [36]. This study is the first to evaluate cellular performance of human AuCPCs after a bioprinting process and to apply these cells for the fabrication of a human ear-shaped construct. Our results indicate that extrusion of AuCPCs through a microvalve system does not negatively affect cell viability, metabolic activity and glycosaminoglycan production. Over the course of 10 days, cell viability was at least 98% and metabolic activity did not differ between cells that were either cast or printed. Similarly, no significant difference was observed in the production of glycosaminoglycans between cast and printed groups after 28 days *in vitro*. Nevertheless, we did observe differences in performance between individual donors. This donor-to-donor variance is a well-known challenge in cells from human sources [85] and would require the characterization of a quality control system to predict the cells’ regenerative potential and thus their usability for clinical application. We elected to fabricate the proof-of-concept auricular module with pediatric AuCPCs, as these cells can be obtained from the healthy contralateral ear in unilateral microtia patients. As AuCPCs can be expanded to high cell numbers from only a low amount of starting material, a small biopsy would be sufficient to provide adequate cell numbers for the fabrication of a full-sized engineered auricular construct. Pediatric AuCPCs from the healthy contralateral ear would constitute a clinically relevant autologous cell source, avoiding risks of implant rejection, while at the same time minimizing donor site morbidity. The fabrication and *in vitro* culture of the auricular module was carried out using a well-performing donor from the pool of studied pediatric AuCPCs, in terms of cartilage regeneration. Abundant glycosaminoglycan production throughout the auricular construct was observed after 30 days *in vitro* culture, as visualized through both histological and contrast-enhanced μCT analysis. The latter technique allows for both qualitative and quantitative analysis. Higher HU values signify a higher concentration of the CA4+ contrast agent, which in turn indicates an increase in GAG content. The application of this novel technique allows for the longitudinal evaluation of GAG production in a non-destructive manner and without hampering chondrogenesis [86]. It provides an opportunity for monitoring engineered auricular constructs preimplantation. Upon moving tissue engineered constructs toward the clinic, non-destructive and non-disruptive evaluation methods are essential in providing a quality control check before deciding that the tissue engineered construct can be implanted into the patient. The proof-of-concept auricular structure presented in this study demonstrates the feasibility of creating hybrid ear-shaped constructs with inherent regenerative potential, as was evidenced by the abundant production of glycosaminoglycans after only 30 days *in vitro* preculture.

High shape fidelity of ear-shaped constructs was observed directly after fabrication and was maintained during the *in vitro* preculture period. The used hybrid bioprinting technique offers a reliable method for the fabrication of an ear shape with a distinct internal architecture. During the fabrication procedure, we observed occasional leakages of the highly aqueous hydrogel from the scaffold before crosslinking. For future studies, this could be prevented by printing a shell of sacrificial materials around the ear-shaped scaffold [34]. Nevertheless, only minor deviations from the digital model were observed that were well below a margin of 1.5 mm elected by Zhou et al. [15]. As hydrogel filling of the ear-shaped scaffold was not perfect, manual correction was applied that influences the results but does not impact deformation outcomes of the scaffold. With an average deviation of 0.21 mm, it can be concluded that shape and size were preserved throughout the dynamic *in vitro* culture period, indicating that the PCL fibers provide adequate construct stabilization during preculture before implantation.

## 5 Conclusion

The engineering of auricular cartilage constructs remains challenging due to the lack of regenerative cells and limited mechanical integrity. Hence, a combination of various bioprinting and cell-based strategies are needed to improve the regenerative performance of engineered tissue products. Although further optimization of the reinforcing scaffold, the printing process, and the culture method may be required, this successful proof-of-concept supports future application of biofabrication-based auricular cartilage engineering. The dual-printed hybrid ear-shaped constructs exhibited excellent shape fidelity during preculture, unaffected cellular performance after printing, and abundant cartilage-like matrix deposition throughout the constructs. Our strategy of combining fiber reinforcement, anatomical enhancement and chondrogenically potent AuCPCs provides an exciting avenue for the development of clinically translatable regenerative strategies for ear reconstruction.

## CRediT Author Statement

**I.A. Otto**: Conceptualization, Methodology, Resources, Investigation, Formal Analysis, Writing – Original Draft, Writing – Review & Editing, Visualization **P.E. Capendale**: Investigation, Formal Analysis **J.P. Garcia**: Validation, Investigation, Formal Analysis, Visualization **M. de Ruijter**: Methodology, Validation, Investigation **R.F.M. van Doremalen**: Methodology, Investigation, Formal Analysis, Visualization **M. Castilho**: Methodology, Supervision **T. Lawson**: Resources **M.W. Grinstaff**: Resources **C.C. Breugem**: Conceptualization, Supervision **M. Kon**: Conceptualization, Supervision **R. Levato**: Conceptualization, Supervision, Writing – Review & Editing **J. Malda**: Conceptualization, Supervision, Writing – Review & Editing

## Ethical statement

All tissues were obtained from biopsies of redundant tissue excised during surgery or from deceased donors who had donated their body to science, based on the guidelines of the Ethical Committee of the University Medical Center Utrecht.

## Data availability

The raw and processed data required to reproduce these findings are available upon request from the corresponding author.

## Declaration of competing interest

The authors declare that they have no known competing financial interests or personal relationships that could have appeared to influence the work reported in this paper.
